# WNT/β-Catenin Signaling Pathway Regulating T Cell-Inflammation in the Tumor Microenvironment

**DOI:** 10.3389/fimmu.2019.02293

**Published:** 2019-09-26

**Authors:** Xin Li, Yanwei Xiang, Fulun Li, Chengqian Yin, Bin Li, Xisong Ke

**Affiliations:** ^1^Department of Dermatology, Yueyang Hospital of Integrated Traditional Chinese and Western Medicine, Shanghai University of Traditional Chinese Medicine, Shanghai, China; ^2^Institute of Dermatology, Shanghai Academy of Traditional Chinese Medicine, Shanghai, China; ^3^Department of Pharmacology & Experimental Therapeutics, Boston University School of Medicine, Boston, MA, United States; ^4^Center for Chemical Biology, Institute of Interdisciplinary Integrative Medicine Research, Shanghai University of Traditional Chinese Medicine, Shanghai, China

**Keywords:** WNT/β-catenin signaling pathway, T cell-inflammation, tumor microenvironment, CD8^+^ T cells, immune exclusion, immunotherapy

## Abstract

Immunotherapy with checkpoint inhibitors has greatly prolonged the overall survival of cancer patients in melanoma and many other cancer types. However, only a subset of patients shows clinical responses from these interventions, which was predicated by the T cell-inflamed tumor microenvironment. T cell-inflamed phenotype is characterized by the infiltration of CD8^+^ T cells, CD8α/CD103-lineage dendritic cells (DCs), as well as high density of forkhead box P3 (FoxP3)^+^ regulatory T cells (Tregs) that are associated with the efficacy of immune checkpoint blockade. A number of regulators has been associated with T cell-inflammation in the tumor microenvironment, and WNT/β-catenin signaling is one of the best characterized. The tumor-intrinsic WNT/β-catenin signaling activation is frequently associated with poor spontaneous T cell infiltration across most human cancers. In this article, we review the essential roles of WNT/β-catenin signaling in the T cell-inflamed and non-T cell-inflamed tumor microenvironment, including the development and function of immune cells, activation of immune exclusion of tumor cells, and cancer immunosurveillance. We also discuss the impact of this pathway in driving the non-T cell-inflamed tumor microenvironment in other tumor types. To improve immunotherapy efficacy, we argue that targeting Wnt/β-catenin signaling should be a high priority for combinational cancer therapy to restore T cell infiltration.

## Introduction

Immunotherapy with immune checkpoint inhibitors (ICIs) is now common for melanoma and many other cancer types (1). An important property of the immune system is to distinguish “foreign” cells from normal cells in the body, which allows the body to attack foreign cells while retaining normal cells. Therefore, it uses “checkpoints”; specifically, molecules on certain immune cells need to be activated (or inactivated) to initiate an immune response (2). As a checkpoint protein on T cells, programmed death-1 (PD-1) normally acts as a type of “off switch” to ensure that T cells do not target other cells in the body (3). Upon binding to PD-L1, which is a ligand protein expressed by some normal (and cancer) cells, it signals T cells to abstain from attacking. Antibodies that target either PD-1 or PD-L1 block this binding and boost the immune response against cancer cells (2). The therapeutic targeting of this axis has shown impressive activity in various tumors including melanoma of the skin, non-small cell lung cancer, head and neck squamous cell carcinoma, renal cell carcinoma, urothelial carcinoma, Hodgkin lymphoma, Merkel cell carcinoma, and microsatellite unstable or mismatch repair-deficient tumors (2). CTLA-4 is another type of checkpoint inhibitor, and monoclonal antibodies that target this marker can also boost the body's immune response against cancer cells (4). For ICIs, and especially CTLA-4 antibodies, one concern is their side effects in some patients as they permit the immune system to attack some normal organs in the body (4).

Despite improvements in clinical outcomes, only the minority of patients responds to ICIs (5). In patients susceptible to immunotherapy, an active immune response is usually observed prior to treatment, which is characterized by infiltrating antigen-specific T cells (6). This phenotype has been described as a T cell-inflamed tumor microenvironment (TME) and can be used to predict responding and non-responding tumors (7). The TME is the environment surrounding the tumor and consists of peripheral blood vessels, immune cells, fibroblasts, signaling molecules, and extracellular matrix (8). Tumors are closely related to the surrounding microenvironment and interact with it continually. Specifically, by releasing extracellular signals, promoting tumor angiogenesis, and inducing peripheral immune tolerance, tumors can affect the microenvironment; in turn, the growth and evolution of cancer cells are also affected by immune cells in the microenvironment (9). In the TME, chemokines support the influx of CD8^+^ effector T cells, which subsequently are functionally inhibited by PD-L1, IDO, Treg cells, and anergy. The development of TME is promoted in part by type I interferon signaling and the CD8a^+^ dendritic cell (DC) lineage (10). In normal tissue microenvironments, chemokine expression is poor and T cell infiltration is lacking, and this is also associated with the minimal presence of defined immune inhibitory pathways (11). In non-T cell inflammatory tumors, T cell inflammatory gene expression is significantly lower than that in matched normal tissues, which is associated with the loss of the native immune phenotype (6). Notably, a recent TCGA database study found that most tumor types are inversely associated with a T-cell-inflamed gene expression signature, and that WNT/β-catenin pathway activation is one potential causal pathway (6).

A correlation between WNT/β-catenin signaling and the T-cell-inflamed TME has been established (6). Moreover, identification of molecular factors associated with non-T cell-inflamed TME is important for the development of combinational immunotherapy regimens. Mechanistic studies based on genetically-engineered mice have also shown that activation of tumor cell-intrinsic β-catenin prevents spontaneous T-cell priming and infiltration into the TME, rendering cells resistant to combinational ICI therapy (12, 13). Thus, pharmacologically targeting the WNT/β-catenin pathway could potentially improve the efficacy of immunotherapy.

Here, we review the mechanisms wherein WNT signaling interrupts immune functions in the T cell-inflamed and non-T cell-inflamed TME, including the development and function of immune cells, activation of immune exclusion in tumor cells, and cancer immunosurveillance. We also discuss the therapeutic potential of harnessing currently available WNT modulators to augment cancer immunotherapy.

## Cancer Immunotherapy and the TME

The T cell-inflamed subset of tumors is dominated by T cell markers and chemokines, which might mediate effector T cell recruitment (14–16). The expression of chemokines C-C motif chemokine ligand (CCL) 2, CCL3, CCL4, CCL5, C-X-C motif chemokine ligand (CXCL) 9, and CXCL10 is associated with the infiltration of T cells, and each of these chemokines is sufficient to recruit CD8^+^ effector T cells *in vitro* (16). Previous studies have confirmed that the T cell-inflamed subset contains variable numbers of CD8^+^ T cells and CD8α/CD103-lineage DCs, but also possesses the highest density of FoxP3^+^ regulatory T cells (Tregs) (16). Additionally, many conventional T cells have a dysfunctional anergic phenotype. It has been found that CXCR3-binding chemokines (such as CXCL9 and CXCL10) are critical and essential for the recruitment of activated CD8^+^ T cells to tumor sites (17). As a major driver of Treg recruitment, CCL22 is partially produced by activated CD8^+^ T cells (18). Despite the presence of specific adaptive immunity in this subset of patients, the cause of tumor progression is likely secondary to immunosuppressive mechanisms that act to some extent in the TME (19). Furthermore, T cell dysfunction in the TME is antigen-specific and restricted to tumor reactive T cells (19).

In contrast, T cell markers and chemokines that mediate T cell recruitment in the non-T cell-inflamed TME are lacking. Macrophages, vascular endothelial cells, fibroblasts, extracellular matrices, and immature DCs in some cases are still present in these tumors (20–24). Moreover, both the priming and effector phases of the anti-tumor immune response are deficient in non-T cell inflammatory tumors (19). Effector T cell trafficking into the TME is complex and dependent on adhesion molecules and homing receptors on vascular endothelial cells, consistent with the fact that chemokines are produced by tumor cells and stromal cells within the TME (19). In most cases, this process is necessary for the clinical response of immunotherapy.

The T cell-inflamed phenotype is associated with the efficacy of immune checkpoint blockade, whereas non-T cell-inflamed tumors rarely benefit. Recently, a series of studies has linked alterations in WNT signaling to oncogenesis, disease progression, and resistance to treatment in the TME (25, 26). Furthermore, dysregulated WNT signaling supports malignant transformation and disease progression through a variety of mechanisms in the TME (27). The high expression of specific immune cell genes in the TME, known as the T-cell-inflamed phenotype, has been associated with response to multiple immunotherapies including therapeutic vaccines and checkpoint blocking antibodies (11, 15, 16, 28–31). In contrast, the non-T-cell-inflamed TME appears to be closely related to a lack of clinical benefit from immunotherapy, particularly in relation to anti-PD-1 antibodies (30, 31). Despite a variety of molecular mechanisms that could be theoretically detrimental to the T-cell-inflamed microenvironment, several studies have indicated that oncogenic molecular aberrations are sufficient to drive the immune exclusion phenotype in some cases (6). In a study using a genetically-engineered mouse model, tumor cell-intrinsic WNT/β-catenin signaling in melanoma was found to be the first somatic alteration associated with the non-T-cell-inflamed TME in patients (13). In addition, the transcriptional repression of key chemokine genes leads to a lack of basic leucine zipper ATF-like transcription factor 3 (Batf3)-lineage DC recruitment, and the subsequent failure to prime and recruit CD8^+^ T cells appears to be involved in this effect (12, 13). This effect is dominant in the TME and results in decreased pre-clinical efficacy for checkpoint blockade, tumor antigen vaccination, and adoptive T-cell transfer immunotherapy approaches (12, 13). In addition, blocking the β-catenin pathway enhances the influx of CD8^+^ T cells and increases IFNγ-related gene targets in syngeneic murine models of B16F10 melanoma, 4T1 mammary carcinoma, Neuro2A neuroblastoma, and Renca renal adenocarcinoma (32). Therefore, strategies to overcome barriers that restrict T cell migration into tumor sites might ultimately promote immunotherapy efficacy in non-T cell-inflamed tumors. The Wnt/β-catenin pathway could thus represent a high-priority target for combinational cancer immunotherapy.

## WNT/β-Catenin Signaling and the Development and Function of Immune Cells

The WNT signaling pathway is highly conserved between species and has been shown to play an important role in controlling multiple developmental processes including asymmetric cell division, stem cell pluripotency, and cell fate specification (33, 34). In addition to the importance of WNT signaling in stem cells and hematopoiesis, its role in the development of T lymphocytes in the thymus is indispensable (35).

T cell factor (TCF), the effector transcription factor of the WNT signaling pathway, was named for its indispensable role in T cell development and proliferation in the thymus (36). The TCF family consists of four members, specifically TCF-1 (encoded by the *TCF-7* gene), TCF-3, TCF-4, and LEF-1 (37, 38). TCF/LEF transcription factors are the major end-point mediators of Wnt signaling throughout metazoans. Although there are other transcription factors that can bind β-catenin and activate transcription, TCFs are major nuclear effector molecules of this pathway (39).

### Wnt/β-Catenin Signaling and CD8^+^ T Cells

CD8^+^ T cells are key effectors of the tumor-immune cycle. In the tumor immune system, CD8^+^ T cells are activated by DCs and co-stimulatory molecules, and then infiltrate into the tumor site to kill target cancer cells (40). By preventing the infiltration of CD8^+^ T cells during tumor progression, tumor cells evade immune elimination, excluding or inactivating CD8^+^ T cells (41). Owing to prolonged antigen exposure and the suppressive TME, tumor-infiltrating CD8^+^ T cells affect the progressive loss of effector functions (42). Wnt/β-catenin signaling is essential for T cell differentiation, effector functions, and migration (43). The activation of TCF-1/β-catenin signaling can result in stem cell-like phenotypes resulting in the formation of memory CD8^+^ T cells or differentiation into a Tfh cell-like gene expression profile *in vivo* (44). Naive CD8^+^ T cells differentiate into effector T cells and kill tumor cells in the tumor-immune cycle (45). Moreover, the activation and maintenance of memory T cells is indispensable to maintain anti-tumor immunity. It has been confirmed that Wnt/β-catenin signaling and TCF1 are highly activated and expressed in undifferentiated CD8^+^ T and memory CD8^+^ T cells, and that TCF1 is downregulated when naive CD8^+^ T cells differentiate into effector CD8^+^ T cells (46).

**Figure 1:**
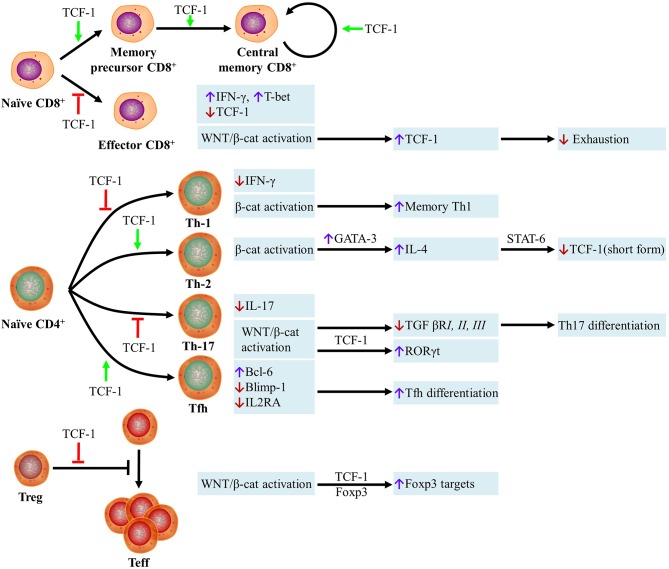
Role of Wnt Proteins in T cells. (i) The differentiation of naïve CD8^+^ T cells into CD8^+^ T effector cells is inhibited, the development of memory precursor and central memory CD8^+^ T cells is promoted, and the expansion is stimulated by TCF-1. Naïve CD8^+^ T cells are resistant to becoming CD8 effector T cells if they are TCF-1^high^ cells, whereas TCF-1^low^ cells become active IFN-γ^+^ T-bet^+^ effector T cells. WNT/β-catenin-mediated activation of effector CD8^+^ T cells can stimulate the transcription of TCF-1, which inhibits type I IFN-mediated cell exhaustion. (ii) The differentiation of naïve CD4^+^ T cells into Th1 and Th17 cells is inhibited and differentiation into Th2 and Tfh subsets is promoted by TCF-1. Unlike TCF-1, all types of T cell differentiation and function are enhanced by the expression of β-catenin. The transcription of TCF-1 is inhibited by T-bet, which is the Th1 hallmark transcription factor, thus preventing the inhibition of T-bet-mediated transcription of IFN-γ. Beta-catenin activation enhances memory Th1 cell formation. In Th2 cells, the transcription of GATA-binding protein-3 (GATA-3) can be induced by β-catenin. GATA-3 stimulates the expression of IL-4, which can bind IL-4R and suppress transcription of the short form of TCF-1 via STAT6. In Th17 cells, IL-17 transcription can be suppressed by TCF-1 directly. Wnt signaling activation inhibits the transcription of TGF-βRI, II, and III, which has an important effect on Th17 differentiation. Upon β-catenin activation, RORγt transcription can be increased by TCF-1 directly. TCF-1 promotes Tfh differentiation by increasing Bcl-6 expression and inhibiting the transcription of Blimp-1 and IL2RA. (iii) TCF-1 inhibits the Treg cell-mediated suppression of effector T cell (Teff) proliferation. In the absence of WNT, the transcriptional repressor Foxp3 inhibits many genes including IL-2. TCF-1 activity is prioritized over Foxp3 when WNT is present, which results in the expression of various transcriptional targets shared by Foxp3 and TCF-1. TCF-1, T-cell factor 1; IFN-γ, interferon gamma; GATA3, GATA binding protein 3; IL-4, interleukin 4; TGFβRI, transforming growth factor beta receptor I; TGFβRII, transforming growth factor beta receptor II; TGFβRIII, transforming growth factor beta receptor III; Blimp-1, B lymphocyte-induced maturation protein-1; IL2RA, interleukin 2 receptor alpha.

Collectively, the differentiation of naïve CD8^+^ T cells into CD8^+^ T effector cells is inhibited, the development of memory precursor and central memory CD8^+^ T cells is promoted, and expansion is stimulated by TCF-1 (Figure 1). However, the requirement for β-catenin in memory T cell functions, as a coactivator of TCF-1, remains controversial.

### Wnt/β-Catenin Signaling and CD4^+^ T Cells

WNT/β-catenin signaling also regulates the differentiation of CD4^+^ helper T (T_H_) cells. TCF-1 and β-catenin support Th2 polarization through activation of the expression of the Th2 master transcription factor GATA binding protein 3 (GATA3) via a special AT-rich sequence binding protein-1 (SATB1), which is a chromatin organizer that plays pivotal roles in T cell development (47, 48). A subsequent study confirmed that the sustained activation of β-catenin in mouse CD4^+^ thymocytes results in the up-regulation of RAR related orphan receptor C (RARC) and consequent Th17 polarization, ultimately resulting in the production of pro-inflammatory cytokines that favor tumorigenesis (49). Another recent study suggested that Wnt10b regulates type 2 inflammation and the activation of Th2 cells, since Wnt10b deficiency was found to exacerbate house dust mite-induced asthma in mice (50). In addition, the selective ablation of TCF-1 or LEF-1 via two plausible mechanisms leads to Tfh deficiency in the LCMV acute infection model (51).

Overall, the differentiation of naïve CD4^+^ T cells into Th1 and Th17 cells is inhibited, whereas differentiation into Th2 and Tfh subsets is promoted, by TCF-1. Further, unlike the effects of TCF-1, all types of T cell differentiation and function are enhanced by the expression of β-catenin (Figure 1).

### Wnt/β-Catenin Signaling and FoxP3^+^ T Cells (Tregs)

It is been shown that the infiltration of Treg cells into the TME results in inhibition of the anti-tumor immune response (41). Treg cells are recruited into the TME by chemokines (such as CCL-28) secreted by tumor cells and innate immune cells (52). Wnt/β-catenin signaling limits the immunosuppressive activity of those cells by modulating the TCF-1-dependent inhibition of FoxP3 transcriptional activity (53). Production of the negative immunomodulators Foxp3, TGFβ, and IL-10 can be reduced by blocking the Wnt/β-catenin signaling pathway in Treg cells (54). Further, if wild-type Treg cells are inactivated, the functional integrity of Tregs will be ensured by the direct activation of genes encoding PD-1 and GITR to increase β-catenin expression, whereas β-catenin levels are reduced and Treg-mediated immune suppression is impaired by liver kinase B1 (LKB1) deletion (55).

In summary, TCF-1 inhibits the Treg cell-mediated suppression of effector T cell proliferation. When WNT is present, TCF-1 activity is prioritized over Foxp3, which results in the expression of various transcriptional targets shared by Foxp3 and TCF-1 (Figure 1).

### Wnt/β-Catenin Signaling and CD8α/CD103-Lineage DCs

The TME regulates DCs through a variety of mechanisms, inhibiting their ability to induce anti-tumor responses. It has been confirmed that the Wnt/β-catenin signaling pathway plays a critical role in crosstalk between tumor cells and DCs within the TME (56). Upon conditional knockout of the Wnt co-receptors LRP5 and LRP6 on DCs, DC-mediated anti-tumor immunity is enhanced, ultimately resulting in delayed tumor growth (57). The recruitment of T cells and DCs into solid tumors is inhibited by β-catenin signaling in melanoma cells (13). In tumor-bearing mouse studies, the activation of β-catenin in DCs resulted in more tolerogenic phenotypes than those mediated by the DC vaccine-induced cross-priming inhibition of anti-tumor CD8^+^ T cells by IL-10 (58). Furthermore, it has been demonstrated that melanoma induces DC-mediated tolerance to inhibit the efficacy of immunotherapy via Wnt5a-mediated mechanisms in mice (59). Notably, DC-specific β-catenin ablation was found to improve anti-PD-1 immunotherapy efficacy in a syngeneic tumor model (59). In antitumor immunotherapy, DCs are crucial subsets that can modulate T cell responses, and studies have summarized the potential to therapeutically target the Wnt pathway in DCs.

The role of the Wnt-β-catenin pathway might be related to the generation of tolerogenic DCs. However, caution is needed before concluding that the Wnt pathway plays a major role in DC function.

### WNT Signaling and Immune Exclusion in the TME

Understanding the mechanisms of T cell exclusion in the TME is critical to improve cancer immunotherapy. The Wnt/β-catenin pathway has been identified as one of the most important oncogenic signaling pathways associated with immune evasion (58, 60). The pan-cancer association between WNT/β-catenin signaling and immune exclusion was previously confirmed by analyzing activated WNT/β-catenin signaling based on somatic mutations, copy number alterations, gene expression, and reverse-phase protein arrays (6). In addition to WNT/β-catenin signaling, other oncogenic events can also contribute to immune exclusion (19, 61). Accordingly, complex interactions among multiple oncogenic events are coupled to mediate more potent immune exclusion in some tumors, such as colorectal cancer, which exhibits the concurrent activation of WNT/β-catenin, MYC, and RAS (62–64).

Mutations in Wnt/β-catenin lead to a non-T cell inflammatory tumor phenotype and might be a biomarker to predict resistance to immunotherapy with ICIs in hepatocellular carcinoma (HCC) (65). Activation of β-catenin reduces T cell infiltrations, increases progression in immunocompetent hosts, and suppresses ICIs in a mouse model of hepatocarcinogenesis (66). The Wnt/β-catenin pathway is also involved in the regulation of innate immunity, such as DCs (67). One study detected an association between activation of Wnt/β-catenin signaling and the loss of T cell gene expression in human metastatic melanoma (13). Beta-catenin activation occurs in 48% of non-T cell inflammatory melanomas (6), and thus, other oncogenic pathways likely contribute to immune exclusion in the remainder of these tumors. Tumor-cell intrinsic activation of the WNT/β-catenin pathway is associated with a lack of T cells in the microenvironment of metastatic melanoma and other cancer types (68). Beta-catenin induces expression of the transcriptional repressor ATF3 and inhibits the transcription of CCL4 in mouse models (69). Furthermore, the defective production of CCL4 results in the impaired infiltration and activation of Batf3-lineage CD103^+^ DCs, reduced CD8^+^ T cell priming and infiltration, and a subsequent lack of responses to immune checkpoint blockade (69). In the absence of active β-catenin signaling, the normal production of CCL4 was found to be restored, resulting in CD103^+^ DC activation and the infiltration and proficient priming of CD8^+^ T cells (69). Wnt/β-catenin signaling within intestinal DCs regulates the balance between inflammatory and regulatory responses in the gut (69). In intestinal DCs, β-catenin was found to be associated with anti-inflammatory mediators, such as retinoic acid-metabolizing enzymes, IL-10, and transforming growth factor-β, as well as Treg-induced stimulation (70).

It has also been shown that Wnt/β-catenin signaling is involved in crosstalk between cancer cells and tumor-associated macrophages. One study demonstrated that interleukin-1β, secreted by tumor-associated macrophages (TAMs), might increase the availability of β-catenin via the phosphorylation of GSK3β in colon cancer cells, thereby disrupting the function of the β-catenin destruction complex (71). Colorectal cancer cells stimulate IL-β production in macrophages via Snail, which is a soluble factor and product of Wnt target genes (72).

**Figure 2:**
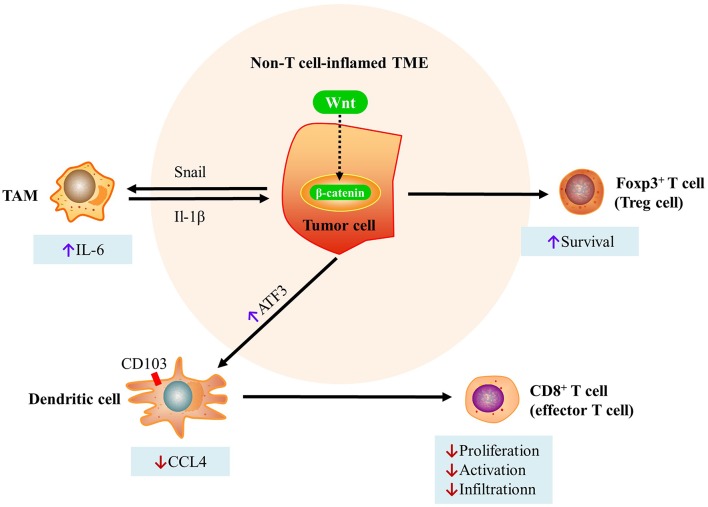
Mechanisms of immune exclusion through the Wnt/beta-catenin pathway. (i) Inhibition of the production of CCL4 in Batf3-lineage CD103^+^ DCs through induction of the expression of the transcriptional repressor ATF3. This in turn reduces the initiation and infiltration of CD8^+^ T cells. (ii) Increases in the interaction between Snail (a soluble factor and product of a Wnt-regulated gene) and TAMs, which in turn increases β-catenin activity via IL-1β. (iii) Enhanced Treg survival [modified from Pai et al. (73)]. DC, dendritic cell; TAMs, tumor-associated macrophages; CCL4, C-C motif chemokine ligand 4; ATF3, activating transcription factor 3; TME, tumor microenvironment.

In summary, the Wnt/beta-catenin pathway mediates immune exclusion via three mechanisms as follows: (i) inhibiting the production of CCL4 in Batf3-lineage CD103^+^ DCs through induction of the expression of the transcriptional repressor ATF3, which in turn reduces the initiation and infiltration of CD8^+^ T cells; (ii) increasing the interaction between Snail (a soluble factor and product of a Wnt-regulated gene) and TAMs, which in turn increases β-catenin activity via IL-1β; (iii) enhancing Treg survival (Figure 2).

## WNT Signaling and Immunosurveillance in the TME

**Figure 3:**
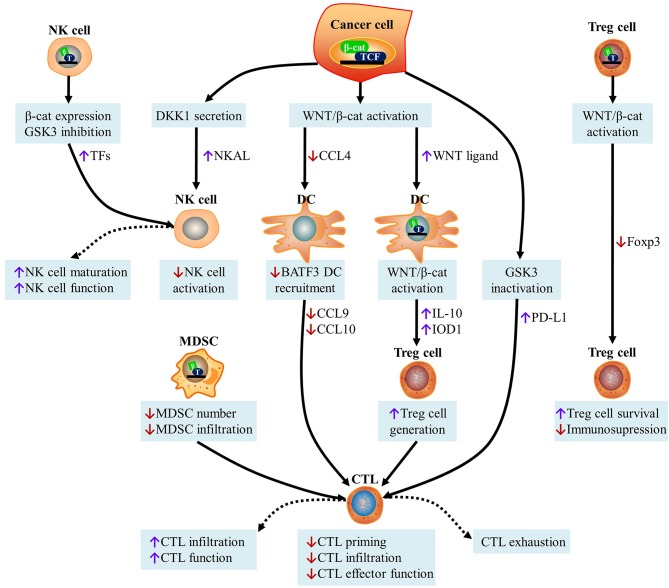
Effect of WNT signaling on cancer immunosurveillance. (i) Activation of the WNT/β-catenin pathway in cancer cells inhibits the secretion of CCL4 and recruits BATF3-dependent DCs to the TME. This causes a decrease in DC-derived CXCL10 levels and limits CD8^+^ CTL infiltration, leading to defective cross-priming. WNT ligands released by cancer cells can induce the canonical WNT signaling pathway in DCs, leading to the increased secretion of IL-10, restricted production of IL-12, and up-regulation of indoleamine IDO1. This contributes to the production of Treg cells and the subsequent inhibition of CTL activity. The inhibition of GSK3 in cancer cells promotes β-catenin activation and subsequently stabilizes PD-L1, which drives CTL exhaustion upon interactions with PD-1. Canonical WNT/β-catenin in MDSCs inhibits their ability to accumulate and infiltrate into malignant lesions, leading to the increased recruitment of CTLs. (ii) DKK1 secreted by latent metastatic cells prevents NK cell-dependent cancer cell lysis, which is caused by the low expression of NKALs, which engage NKARs. GSK3 inhibition and β-catenin overexpression in NK cells lead to the up-regulation of transcription factors responsible for NK cell maturation, thereby stimulating the function of NK cells as effectors. (iii) Although the expression of β-catenin is beneficial for the survival of Treg cells *in vitro*, the activation of TCF-1, downstream of canonical WNT signaling, blocks Foxp3 transcriptional functions and impairs the immunosuppressive activity of Treg cells. CTL, cytotoxic T cell; DKK1, Dickkopf WNT signaling pathway inhibitor 1; NKALs, NK cell activating ligands; NKARs, NK-cell-activating receptors; MDSCs, myeloid-derived suppressor cells; DC, dendritic cell; CCL4, C-C motif chemokine ligand 4; TCF, T cell factor; BATF3, basic leucine zipper ATF-Like transcription factor 3; GSK3, glycogen synthase kinase 3; PD-L1, programmed cell death ligand 1; NK, natural killer; IL-10, interleukin 10; IDO1, indoleamine 2,3-dioxygenase 1; TFs, transcription factors; Foxp3, forkhead box p3.

It has been widely accepted that WNT/β-catenin signaling affects cancer immunosurveillance across cancer types (27). By modulating various aspects of tumor-immune cell interactions including the immunogenicity of cancer cells and the ability of immune cells, such as DCs, natural killer (NK) cells, Treg cells, myeloid-derived suppressor cells (MDSCs), and cytotoxic T lymphocytes (CTLs) to elicit effective tumor-targeting immune responses, it has been discovered that WNT signaling positively or negatively affects anticancer immunosurveillance (Figure 3).

Tumor-intrinsic WNT/beta-catenin signaling affects the immunogenicity of carcinoma cells. Several components of the WNT signal transduction cascade that are overexpressed in carcinoma cells as tumor-associated antigens can be recognized by the immune system (74). Moreover, it has been confirmed that WNT signaling is a major regulator of tumor immune tolerance. Active forms of β-catenin promote immune evasion and resistance to immunotherapy with anti-PD-1, which was found to involve the deficient recruitment of DCs and impaired T cell activity in a novel mouse model of HCC (75). A previous study showed that tumor-induced β-catenin signaling plays a role in DCs, resulting in inhibition of the DC-dependent cross-sensitization of anti-tumor CTLs and infiltrating immune effector cells into a tolerant state (76).

The accumulation of β-catenin in human melanoma cells or DCs leads to the expression of IL-10, which diminishes the capacity of DCs to cross-prime CD8^+^ CTLs (77). In melanomas, canonical or non-canonical WNT signaling also limits antitumor immunity in DCs, which is associated with metabolic immunosuppression by IDO1 (78). This indicates that the activation of this pathway in DCs is important to maintain clonal amplified antigen-specific CTLs, although most studies indicate that classical WNT signaling inhibits antitumor CTL cross-priming. These studies indicate that the role of WNT signaling in the regulation of anticancer responses during CTL activation is stage-dependent (27).

Excluding CTL infiltration into the TME is the prominent mechanism of WNT-mediated immunoevasion in multiple types of cancer. Therefore, the conditional expression of oncogenic Braf plus Pten deletion and the engineered expression of β-catenin in mouse melanomas results in cells that are unable to express CCL4, leading to DC recruitment defects (13). Due to the absence of CD103^+^ DC-derived chemokines, such as CXCL9 and CXCL10, tumor infiltration by CTLs is prevented and antitumor immune responses are impaired (13). It is generally accepted that effector cell deficiency in the TME is associated with active classical WNT signaling, which is also the main cause of resistance to cancer immunotherapy (79). Actually, tumor-intrinsic, active β-catenin signaling in human melanoma causes resistance to monoclonal antibodies including anti-PD-L1and anti-CTLA4 via T cell exclusion (13). In the absence of WNT ligands, GSK3β mediates the phosphorylation-dependent proteasomal degradation of PD-L1 by β-TrCP, and tumor infiltration is ultimately increased by interferon-producing CTLs (80).

WNT signaling exerts significant anticancer effects. For example, limiting β-catenin signaling prevents carcinogenesis and the accumulation of CD11b^+^Gr-1^+^ MDSCs (81). Beta-catenin inhibits the downstream deletion of *Plcg2* (82), *Cul4b* (83), or *Mucl* (84), as well as the increased availability of DKK1 in the microenvironment (85), leading to MDSC amplification and recruitment into the TME, attenuating specific immune responses in different types of tumors. Quiescence in lung metastatic cancer cells is caused by autocrine DKK1, which leads to the downregulation of certain cell surface markers recognized by NK cells, allowing cancer cells to evade innate immunity (86).

In summary, cancer immunosurveillance is influenced by WNT signaling in a complex and context-dependent manner.

## WNT Modulation for Cancer Immunotherapy

The WNT/β-catenin pathway should be a high-priority molecular target for new drug development, in an effort to restore T cell infiltration and potentially expand the efficacy of immunotherapy. Several specific inhibitors have been developed and have entered preclinical trials (Table 1); however, the activity of monotherapy has not been sufficient to warrant further advances to registered trials (87). Beta-catenin signaling is widely utilized by many cell types, and thus on-target, off-tumor effects limit the potential of therapeutics that target this protein. The immune evasive mechanisms of β-catenin activation, including the inhibition of chemokines and cytokine gene expression by tumor cells (13), provide the opportunity to develop agents with more restricted activities that predominantly augment immunity while maintaining other essential cellular functions.

**Table 1:** WNT inhibitors for cancer immunotherapy currently in clinical development.

**Mechanism of action**	**Agent**	**Stage of clinical development**	**Status**	**Details**	**Trial phase; Identifier**
Anti-FZD7 antibody	OMP18R5	Phase 1	Completed	Metastatic breast cancer with locally recurrent or metastatic; in combination with paclitaxel	NCT01973309
AXIN1 activator	Niclosamide	Phase 1	Recruiting	Colon cancer subjected to primary tumor resection	NCT02687009
		Phase 1	Recruiting	Metastatic prostate carcinoma, recurrent prostate carcinoma, and stage IV prostate cancer	NCT03123978
		Phase 2	Recruiting	Metachronous or synchronous metastases during colorectal cancer progression under standard therapy	NCT02519582
AXIN1 activator	XAV939	Preclinical			
COX2 inhibitor	Celecoxib	Phase 2	Withdrawn	Breast carcinoma	NCT03185871
DVL2 inhibitor; PORCN inhibitor	IWP-L6	Preclinical		Favors tumor infiltration by IFNG-producing CD4^+^ and CD8^+^ T cells; depletes intratumoral Treg cells	
FZD10-targeting ARC	OTSA101	Phase 1	Terminated	Doxorubicin and ifosfamide-refractory synovial sarcoma	NCT01469975
FZD8-Fc Decoy receptor	OMP-54F28	Phase 1b	Completed	Locally advanced or metastatic hepatocellular cancer, in combination with sorafenib	NCT02069145
		Phase 1b	Completed	Recurrent platinum-sensitive ovarian cancer, in combination with paclitaxel and carboplatin	NCT02092363
		Phase 1b	Completed	Untreated stage IV metastatic pancreatic cancer, in combination with gemcitabine and nab-paclitaxel	NCT02050178
		Phase 1	Completed	Metastatic and unresectable refractory solid tumors	NCT01608867
PORCN inhibitor	C59	Preclinical		Synergizes with CTLA4-targeting antibodies in mouse melanoma models	
PORCN inhibitor	ETC1922159	Phase 1a/1b	Active, not recruiting	Locally advanced or metastatic solid tumors	NCT02521844
PORCN inhibitor	RXC004	Phase 1	Not yet recruiting	Advanced malignancy not considered appropriate for further conventional treatment	NCT03447470
PORCN inhibitor	WNT974; LGK974	Phase 1/2	Completed	BRAF-mut mCRC and WNT pathway mutations; in combination with LGX818 and cetuximab	NCT02278133
		Phase 2	Withdrawn	Metastatic head and neck squamous cell carcinoma	NCT02649530
		Phase 1	Recruiting	Documented BRAF mut for mCRC and pancreatic cancer; tumors of any histological origin with documented genetic alterations upstream of Wnt signaling	NCT01351103
Unclear	Artesunate	Phase 2	Recruiting	Single primary site colorectal adenocarcinoma or high-grade dysplasia plus unequivocal radiological evidence of invasive cancer	NCT02633098
Unclear	SM08502	Phase 1	Recruiting	Advanced solid tumors who are refractory to or intolerant of established therapy	NCT03355066
WNT decoy	OMP54F28	Phase 1	Completed	Solid tumor with metastasis or unresectable	NCT01608867
WNT inhibitor	CGX1321	Phase 1	Recruiting	Locally advanced or metastatic solid tumors	NCT02675946
		Phase 1	Recruiting	Advanced Gl tumors, such as colorectal adenocarcinoma, gastric adenocarcinoma, pancreatic adenocarcinoma, bile duct carcinoma, hepatocellular carcinoma, esophageal carcinoma	NCT03507998
WNT5A inhibitor	WNT5A trap	Preclinical		Modulates the immunological tumor context; favors doxorubicin-driven immunogenic cell death	
β-Catenin inhibitor	PKF115-584	Preclinical		Restores CTL activation *in vivo*	
β-Catenin inhibitor	PRI724	Phase 1b	Completed	Advanced or metastatic pancreatic adenocarcinoma, in combination with gemcitabine in the second line of treatment	NCT01764477
		Phase 1/2	Completed	Advanced myeloid malignancies	NCT01606579
		Phase 2	Withdrawn	Advanced mCRC; in combination with mFOLFOX6 + bevacizumab, in the first line of treatment	NCT02413853
		Phase 1a/1b	Terminated	Phase 1a: any advanced neoplasm; Phase 1b: only patients with mCRC	NCT01302405

*mCRC, metastatic colorectal cancer; ARC, antibody-radionuclide conjugate; COX2 (official name PTGS2), prostaglandin-endoperoxide synthase 2; DVL2, disheveled segment polarity protein 2; FZD7, frizzled class receptor 7; PORCN, Porcupine O-Acyltransferase; CTLA-4, cytotoxic T-lymphocyte-associated protein 4; CTL, cytotoxic T lymphocyte*.

Over the past few years, a variety of agents that target main components or modulators of WNT signaling have been developed and used as tumor therapies. Different clinical trials listed in the National Library of Medicine database are testing the effectiveness and safety of these drugs, which have not been approved by the regulatory agencies for clinical use. At first, drug development focused on disrupting WNT-driven tumor growth, including monoclonal antibodies to frizzled class receptor (FZD) receptors (e.g., OMP-54F28, OMP-18R5) (88), porcupine O-acyltransferase (PORCN) inhibitors (e.g., WNT974, RXC004, ETC1922159) (89), AXIN1 activators (e.g., Niclosamide, XAV939) (90), and β-catenin inhibitor (e.g., PRI724, PKF115-584) (91). Even though the immunomodulatory effects of these drugs have been neglected for a long time, there is increasing evidence suggesting that WNT inhibitors can also help to reestablish anticancer immunity. For example, the β-catenin inhibitor PKF115-584 effectively stimulates DC suppression, leading to a robust therapeutic response (77). Furthermore, both IWP-L6 and XAV939 deplete Treg cells from the TME, thus achieving a therapeutically-relevant immune response in an animal model of melanoma and lymphoma (57, 92). Various mechanisms associated with the cancer immunomodulatory effects of WNT inhibitors are listed in Table 1.

The immunological context of the TME has an important effect on the clinical efficacy of cancer immunotherapy, including its composition, activation status, and localization (93). Actually, the inhibition of local or systemic immunosuppression is the target of immunotherapy that is used to restore CTL depletion and reinstate the immunological control of tumor growth (94). ICIs limit the capability of co-inhibitory receptors including PD-1 and CTLA4, which are used to establish CTL depletion. However, when CTLs are absent or unable to infiltrate malignant cell niches, the efficacy of immunotherapy with ICIs is severely reduced (95). Since this phenotype is common in tumors that present with WNT activation, inhibitors of canonical WNT signaling can be combined with CTLA4-blocking mAbs to suppress the progression of melanoma in mice (78). Notably, molecular profiling of HCC has identified that patients with ICIs and activating alteration in WNT/β-catenin signaling have lower disease control rates, shorter median progression-free survival, and shorter median overall survival (96). One recent study developed potent, selective inhibitors targeting the interaction of β-catenin/Bcl9, which overcome resistance to ICIs by modulating Treg cells (97).

The activation of anticancer immune responses largely determines the efficacy of several conventional therapeutic strategies for the treatment of cancer, including a variety of chemotherapeutics, radiation therapy, and some targeted anticancer agents (98). Different anticancer molecules, such as oxaliplatin and doxorubicin can result in a noticeable immunogenic variant of cell death to establish adaptive anticancer immunity (99). Further, it has been confirmed that WNT signaling limits the efficacy of multiple anticancer therapies in this manner (10). Accordingly, it has been found that efficient local Wnt5a trapping results in significant remodeling of the immunosuppressive TME and promotes immunogenic cell-death-mediated immunotherapy (10).

The activity of chemotherapy and immunotherapy with ICIs can be boosted by WNT inhibitors. Studies have shown that CD8^+^ T cells and CD4^+^ T_H_17 cells can differentiate into stem-like cells with advantageous anticancer functions upon the intervention with GSK3 inhibitors (46, 100); they also stimulate NK cell-dependent anticancer immunity (86). The restoration of β-catenin expression in MDSCs and the use of an anti-DKK1 mAb also limit tumor growth by establishing a TME that is beneficial for immunocompetency (85). Therefore, based on the existing preclinical evidence, WNT activation can constitute a therapeutic target.

## Conclusion and Future Prospects

This review highlights the role of WNT signaling in tumor initiation, malignant tumor progression, and resistance to therapeutics. In the context of combinatorial treatment regimens, a promising candidate WNT modulator, to improve the efficacy of various immunotherapeutic agents, is currently being validated in a variety of preclinical tumor models.

Many preclinical studies have demonstrated that the efficacy of ICIs is affected by WNT/β-catenin signaling, which reduces the infiltration of CTLs into the TME. In contrast, the insensitivity of melanoma patients to immunotherapy with ICIs affects the expression levels of certain components of the WNT signaling cascade (3). In terms of the future direction, it is important to identify biomarkers associated with the non-T cell inflammatory tumor microenvironment. Although WNT modulators are in development for use in combination immunotherapy regimens for cancer, further preclinical and clinical studies are needed to confirm their utility.

## Author Contributions

XK and XL conceived and wrote this manuscript. XL, YX, FL, CY, BL, and XK discussed the literature and contributed to the revision of the manuscript. CY contributed to the revision of the manuscript. All authors have read and approved the final manuscript.

### Conflict of Interest

The authors declare that the research was conducted in the absence of any commercial or financial relationships that could be construed as a potential conflict of interest.
